# Clock drawing test strategies among older adults with and without cognitive impairment

**DOI:** 10.1590/1980-5764-DN-2025-0458

**Published:** 2026-08-24

**Authors:** Bárbara Spenciere, Liana Chaves Mendes dos Santos, Helenice Charchat-Fichman

**Affiliations:** 1 Pontifícia Universidade Católica do Rio de Janeiro, Departamento de Psicologia, Rio de Janeiro, RJ, Brazil.

**Keywords:** Dementia tests, Executive Functions, Alzheimer Disease, Cognitive Dysfunction, Strategic Planning., Testes de Estado Mental, Função Executiva, Doença de Alzheimer, Disfunção Cognitiva, Planejamento Estratégico.

## Abstract

In addition to being used as a screening test, the Clock Drawing Test (CDT) has been used to evaluate executive functioning in older adults. Using a new validated scoring method that classifies CDT construction — planning and organizing strategies —, published in a previous article, the strategies used to perform the test in older adults with cognitive impairment were investigated.

**Objective:**

Describe and classify the planning strategies used by older adults with cognitive impairment to perform the CDT, using the CDT Strategies scoring method.

**Methods:**

The study included 86 older adults, divided into three groups: 45 with mild cognitive impairment, 13 with major cognitive impairment, and 28 controls.

**Results:**

The analysis of the results emphasized CDT strategies and the neuropsychological profile of older adults. The subcategories associated with better executive functioning decrease in occurrence as cognitive impairment becomes more pronounced, and the subcategories associated with worse executive functioning increase in frequency.

**Conclusion:**

The qualitative analysis proposed by CDT Strategies has clinical utility as a measure of executive functioning.

## RESUMO

Além de ser usado como triagem, o Teste de Desenho do Relógio (TDR) tem sido utilizado para avaliar o funcionamento executivo em idosos. Com base em um novo método de pontuação validado que classifica a construção do TDR — estratégias de planejamento e organização, publicado em um artigo anterior, investigaram-se as estratégias usadas para realizar o teste em idosos com comprometimento cognitivo.

**Objetivo:**

Descrever e classificar as estratégias de planejamento utilizadas por indivíduos idosos com comprometimento cognitivo para realizar o TDR, usando o método de pontuação das Estratégias do TDR.

**Métodos:**

O estudo incluiu 86 idosos, divididos em três grupos: 45 com comprometimento cognitivo leve, 13 com comprometimento cognitivo maior e 28 controles.

**Resultados:**

A análise dos resultados enfatizou as estratégias de planejamento do TDR e o perfil neuropsicológico dos idosos. As subcategorias associadas a um melhor funcionamento executivo diminuem em ocorrência à medida que o comprometimento cognitivo se torna mais acentuado e as subcategorias associadas a um pior funcionamento executivo aumentam em frequência.

**Conclusão:**

A análise qualitativa das Estratégias do TDR possui utilidade clínica como medida do funcionamento executivo.

## INTRODUCTION

The Clock Drawing Test (CDT) has been used over the past 30 years for the early screening of cognitive impairment, particularly in dementia. CDT is a nonverbal neuropsychological instrument that can be easily administered. As a screening test with a wide range of cognitive domains, CDT can also be used for more specific analyses, especially executive functioning, when the focus is on the planning and organization used to perform the task.

There are many ways to score CDT^1,2^. Equivalences between distinct scoring methods have been identified^1^, and studies have concluded that the different methods accurately discriminate between normal and pathological aging^2,3^. The analyses of scoring methods can be classified as quantitative, semi-quantitative, and qualitative^4,5^. Recently, qualitative analyses have become more widely used^6,7,8,9^.

A numerical scoring system that considers planning and organization and focuses on describing the sequence of the drawing construction process can provide more information about cognitive functioning^10,11^. The joint use of quantitative and qualitative scoring systems has been referenced as important in increasing the clinical usefulness of the instrument^12^, including digital or automated systems of the CDT^13^.

Considering these aspects, CDT^11^ Strategies were created. This is a new classification system for CDT scoring based on planning strategies. From the analysis of videos of older adults drawing the clock during administration of the CDT, the strategies used were classified and categorized, considering the sequential process of making the drawings^2,11^. The construct validity of this scoring system was carried out with participants without cognitive impairment from a heterogeneous community^2,11^. Associations were found between CDT strategies and sociodemographic data, types of error on the CDT qualitative scale^6^ and associations with classical cognitive paradigms that assess executive functioning, with the following instruments: Rey Complex Figure Test (RCFT)^14^, Construction — Mattis Dementia Rating Scale (MDRS)^15^, Block Design Test (BDT) Wechsler Adult Intelligence Scale (WAIS-III)^16^, Corsi Block Test (CBT)^17^.

This study aimed to describe and classify the planning strategies used by older adults with cognitive impairment to perform CDT, using the CDT Strategies scoring method. Another objective was to compare these strategies with those previously described by older adults without cognitive impairment^2,11^.

## METHODS

### Participants

The study included 86 community-dwelling older adults (66W; 20M) aged 60–90 years (M=73.87, standard deviation — SD=7.47) and schooling (M=11.48, SD=5.39) who attended a program provided by the city of Rio de Janeiro. They were divided into a control group (28 without cognitive impairment) and a clinical group (45 mild cognitive impairment — MCI), 13 major cognitive impairment — MjCI). The MjCI were: six Alzheimer’s disease (AD) and seven mixed dementia. The MCI subtypes are classified according to the Barbosa et al.^18^ classification algorithm: Amnestic MCI-Single Domain (MCI-A: 18), Amnestic MCI-Multiple Domain (MCI-A MD: 17), and Non-Amnestic MCI Single Domain (MCI N-A: 10). The clinical diagnoses were made by a psychiatrist, including the neuropsychological testing outcomes, according to DSM-5. The psychiatrist has not used CDT in clinical diagnosis; he did not have access to the participants’ CDT results.

The inclusion criteria were corrected visual or auditory deficits and more than four years of schooling. Nine participants were excluded from the analysis (major depression, history of bipolar disorder, schizophrenia, substance abuse, traumatic brain injury, stroke, exposure to neurotoxic substances or brain tumors).

This research was approved by the Ethics Committee of Plataforma Brasil (Opinion no. 965.264, Certificate of Presentation for Ethical Appreciation — CAAE: 39381514.3.0000.5285). All individuals signed an informed consent form before data collection.

### Instruments and procedures

All participants underwent a functional and cognitive evaluation: Brief Cognitive Screening Battery^19^; MDRS^15^; Rey Auditory Verbal Learning Test (RAVLT)^20^; Geriatric Depression Scale (GDS-15)^21^; Pfeffer Functional Activities Questionnaire (PFAQ)^22^, and CDT.

The CDT administration method utilized was Sunderland et al.^23^. The drawings were analyzed using semi-quantitative analysis^23,24^ (Supplementary Material – available at https://www.demneuropsy.org/wp-content/uploads/2026/06/DN-2025.0458-Supplementary-Material.docx), qualitative analysis^6,8^ (Supplementary Material), and the CDT Strategies method.

CDT Strategies is a new, validated scoring method that classifies CDT construction — planning and organizing strategies^11^. The CDT was filmed for analysis of the strategies used to perform drawing. The clinical groups’ videos were analyzed, and two new categories (Mixed-General Sequence and Atypical-Numerical Sequence) were added to include patterns of strategies. The criterion of highest frequency was based on the description and categorization. The final categories were (examples in https://www.demneuropsy.org/wp-content/uploads/2026/06/DN-2025.0458-Supplementary-Material.docxSupplementary Material):

General Sequence: Circle-number-center-hand; Circle-number-hand; Circle-center-number-hand; Atypical – the person does not follow any of these categories; and Mixed — the person changes the strategy during the draw.

Number Sequence: Sequential — the numbers are placed following the clockwise and counterclockwise orders; Quadrant — the person first places numbers in the quadrants 12, 3, 6, 9, and then the other numbers; Half — the person first places numbers in the median line of the clock and then the other numbers in ascending (12,6,1,2,3,4,5,7,8,9,10,11); Mixed-Subtype — the person places the numbers initially in one strategy and changes during the draw; Mixed-Subtype Sequential-Quadrant (12,1,2,3,6,9,4,5,7,8,10,11); Mixed-Subtype Sequential-Half (12,1,2,3,6,4,5,7,8,9,10,11); Mixed-Subtype Sequential-Sequential (12,1,2,3,11,10,9,8,7,6,5,4); Mixed-Subtype Half-Quadrant (12,6,1,2,3,4,5,9,7,8,10,11); Atypical — when numbers are absent.

**Figure 1:**
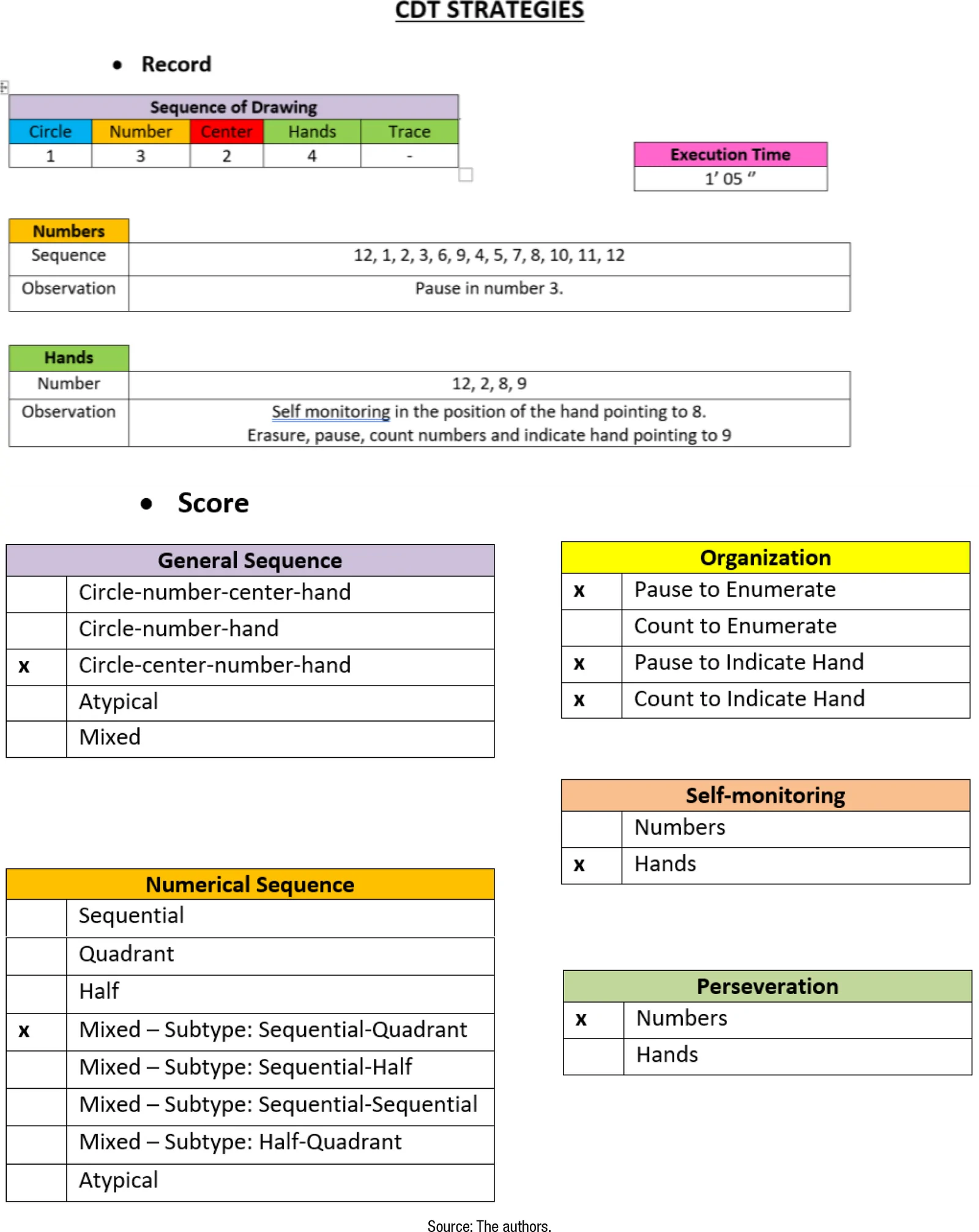
Example of a record and score sheet filled out.

Source: The authors.

Figure 1 shows an example of registering the drawing analysis of a participant with MCI. The sequence of the drawing was registered during the drawing (e.g., first the circle, secondly the center, then the numbers, and finally the hands). While the participant was writing the numbers, the sequence of the numbers was registered (e.g., 12-1-2-3-6-9-4-5-7-8-10-11-12). When he was writing number 3, he paused the drawing and changed the strategy of drawing. So, this observation was written down. While hands were made, the numbers the hands were pointed to were registered. An observation of the self-monitoring behavior of the participant was registered. Finally, the type of score was chosen in each category.

Also, three items were added to the Record and Score Sheet from the preceding study^11^: Execution time (time spent to draw the clock); Organization and self-monitoring (Numbers: attempt to correct the positioning of the numbers on the clock and/or the change in planning strategy in numerical sequence/ Hands: presence of self-corrections in an attempt to position hands correctly); and Perseverence (record of more than two hands that were not motivated by monitoring the plan to better achieve the objective of correctly positioning the hand). They were registered during the clock drawing process. All those elements will be explored in future studies.

### Data analysis

For data analysis, quantitative and qualitative analyses were performed. Statistical analyses were performed in the Statistical Package for the Social Sciences (SPSS) 20.0 version, p≤0.05 (5%). The sample was characterized by descriptive statistical analysis. The Kolmogorov-Smirnov test was used, and the variables did not have a normal distribution. Then, Spearman’s correlation was used. The non-parametric Kruskal-Wallis test was used to compare the differences between control and clinical groups regarding the scoring methods of the CDT, measures of cognitive functioning, age, and educational level. Additionally, a chi-square test was performed to analyze group differences for categorical variables. Following the inferential analyses, the discrepancy between observed and expected frequencies of association between CDT strategies was verified by chi-square analyses. Adjustments were made when the comparison cells had 20% of the observations lower than 5. This analysis was chosen as a direct group comparison, because CDT strategies are categorical variables and, in this way, could be better described in subtle aspects.

## RESULTS

The Kolmogorov-Smirnov test was used to verify if variables had a normal distribution. Spearman’s correlation was used for the semi-quantitative and qualitative CDT, and the results showed a significant and positive correlation (r=0.676, p<0.01) in the control group as well as for the group with MCI (r=0.751, p<0.01). On the other hand, the correlation was not significant (r=0.339, p=0.258) for the group of older adults with MjCI.

In terms of education level, no statistically significant differences were found between the groups. On the other hand, significant differences were present between all groups regarding age (Table 1).

**Table 1:** Sociodemographic characteristics, neuropsychological and functional profiles, and Clock Crawing Test performance of the control group, participants with mild cognitive impairment, and participants with major cognitive impairment.

n	Control	MCI	MjCI	p-value
	28	45	13	
Gender (female/male)	25/3	33/12	8/5	<0.001
Age	72.29 (6.87)	73.87(8.07)	77.31(5.6)	=0.58*
				=0.10^†^
				=0.13^‡^
Education (years)	14.11 (5.05)	10.82 (4.8)	8.08 (5.4)	=0.01*,^†^
				=0.005^‡^
MMSE	26.61 (2.74)	24.73 (2.23)	18.23 (4.85)	=0.01*
				<0.001^†,‡^
Verbal Fluency -Animals	19.46 (4.52)	14.82 (5.2)	10.08 (5.75)	<0.001*,^‡^
				=0.06^†^
MDRS Total	139.03 (3.96)	124.91 (11.41)	98.30 (21.02)	<0.001*,^†,‡^
RAVLT - Sum A1-A5	47.53(10.1)	34.68 (9.28)	20.07(7.38)	<0.001*,^†,‡^
RAVLT - A7	9.71 (3.01)	4.46 (3.27)	1.07(1.65)	<0.001*,^‡^
				=0.01^†^
GDS-15	3 (2.35)	3.7 (2.93)	3.77 (1.9)	0.17*
				0.80^†^
				0.35^‡^
PFAQ	0.38 (1.38)	1.32 (1.63)	16.23 (7.94)	<0.001^†,‡^ =0.002*
CDT semi-quantitative23	6.1(2.1)	5.35 (1.86)	3.84(0.98)	=0.47*
				=0.01^†,‡^
CDT qualitative6	13.17 (1.72)	12.17 (1.82)	10.0 (1.58)	=0.10*
				<0.001^‡^
				<0.01^†^

Abbreviations: MCI, mild cognitive impairment; MjCI, major cognitive impairment; MMSE, Mini-Mental State Examination; MDRS, Construction – Mattis Dementia Rating Scale; RAVLT, Rey Auditory Verbal Learning Test; GDS, Geriatric Depression Scale; PFAQ, Pfeffer Functional Activities Questionnaire; CDT, Clock Drawing Teste.

Notes: *Control x MCI;

^†^MCIxMjCI;

^‡^ControlxMjCI.

Performance in the semi-quantitative CDT^23,24^ showed a higher frequency of score 5 for control (F=13, 46.4%) and MCI (F=21, 46.6%). For the MjCI, the same frequency was obtained (F=4, 30.7%) of scores 3, 4, and 5.

Regarding the effect of cognitive impairment on the results of the semi-quantitative and qualitative CDT, significance was observed between the control and MjCI and between MCI and MjCI (p<0.01), but not between MCI and control.

### Clock drawing teste strategies

Based on the analysis of the videos, specific patterns of drawing strategies in participants with cognitive impairment were observed in comparison with the previously categorized strategies in older adults without cognitive impairment^11^. The new categories added were: Mixed (General Sequence) and Atypical (Numerical Sequence).

**Figure 2:**
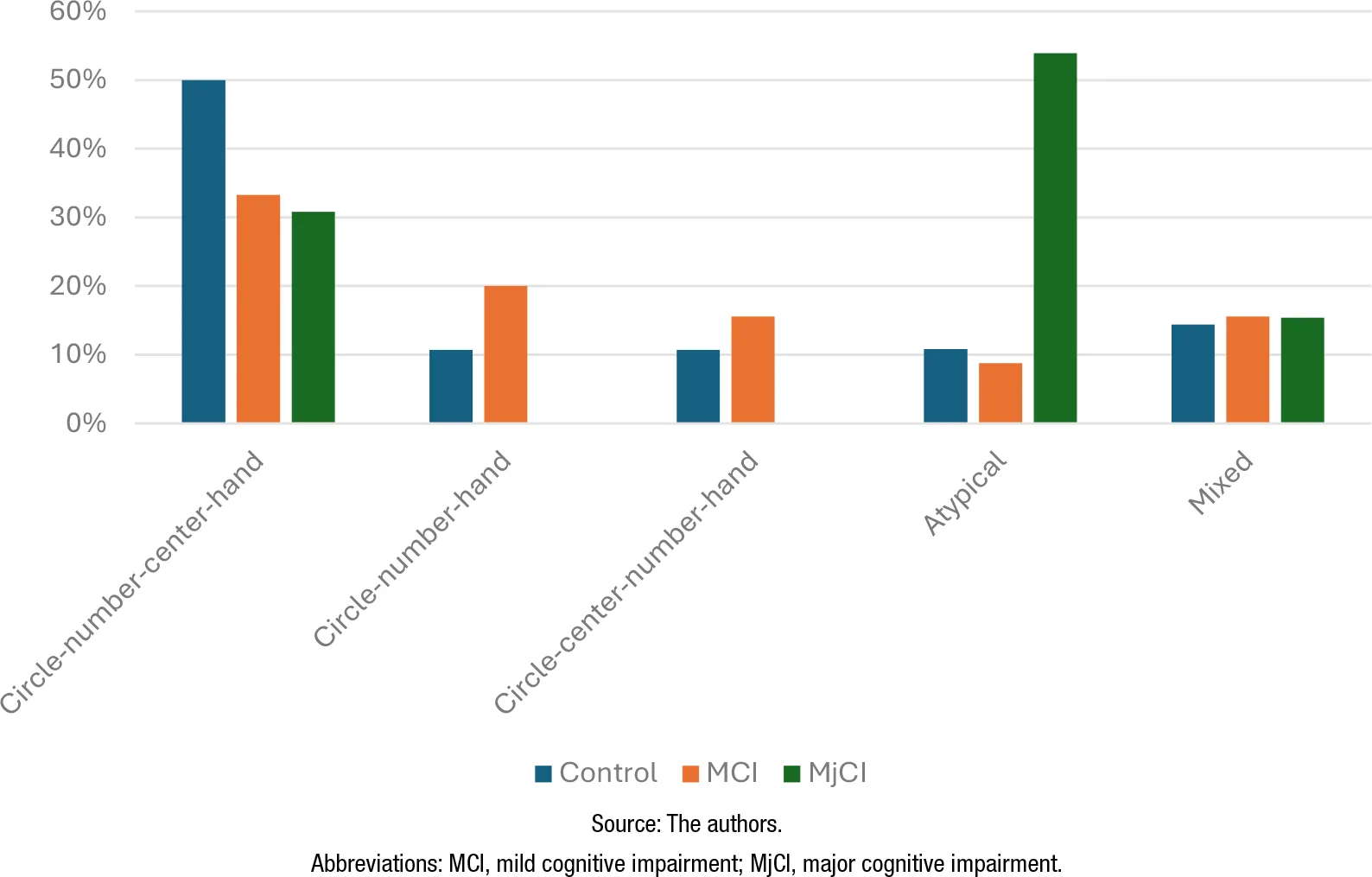
Distribution ratio of general sequence strategies.

Source: The authors.

Abbreviations: MCI, mild cognitive impairment; MjCI, major cognitive impairment.

Regarding the General Sequence (Table 2), the control and MCI showed a higher frequency of the Circle-number-center-hand. There is also a decrease in its occurrence as cognitive impairment intensifies. On the other hand, there is an increase in the frequency of the Atypical in the MjCI group compared to the others (Figure 2).

**Table 2:** Discrepancy between observed and expected frequency of association between Clock Drawing Teste Strategies — General Sequence and neuropsychological profile.

n	Frequency	Control	MCI	MjCI
		28	45	13
General sequence				
Circle-number-center-hand	Observed	14	15	4
	Expected	10.7	17.3	5
Circle-number-hand	Observed	3	9	0
	Expected	3.9	6.3	1.8
Circle-center-number-hand	Observed	3	7	0
	Expected	3.3	5.2	1.5
Atypical	Observed	4	7	7
	Expected	5.9	9.4	2.7
Mixed	Observed	4	7	2
	Expected	4.2	6.8	2

Abbreviations: MCI, mild cognitive impairment; MjCI, major cognitive impairment.

**Figure 3:**
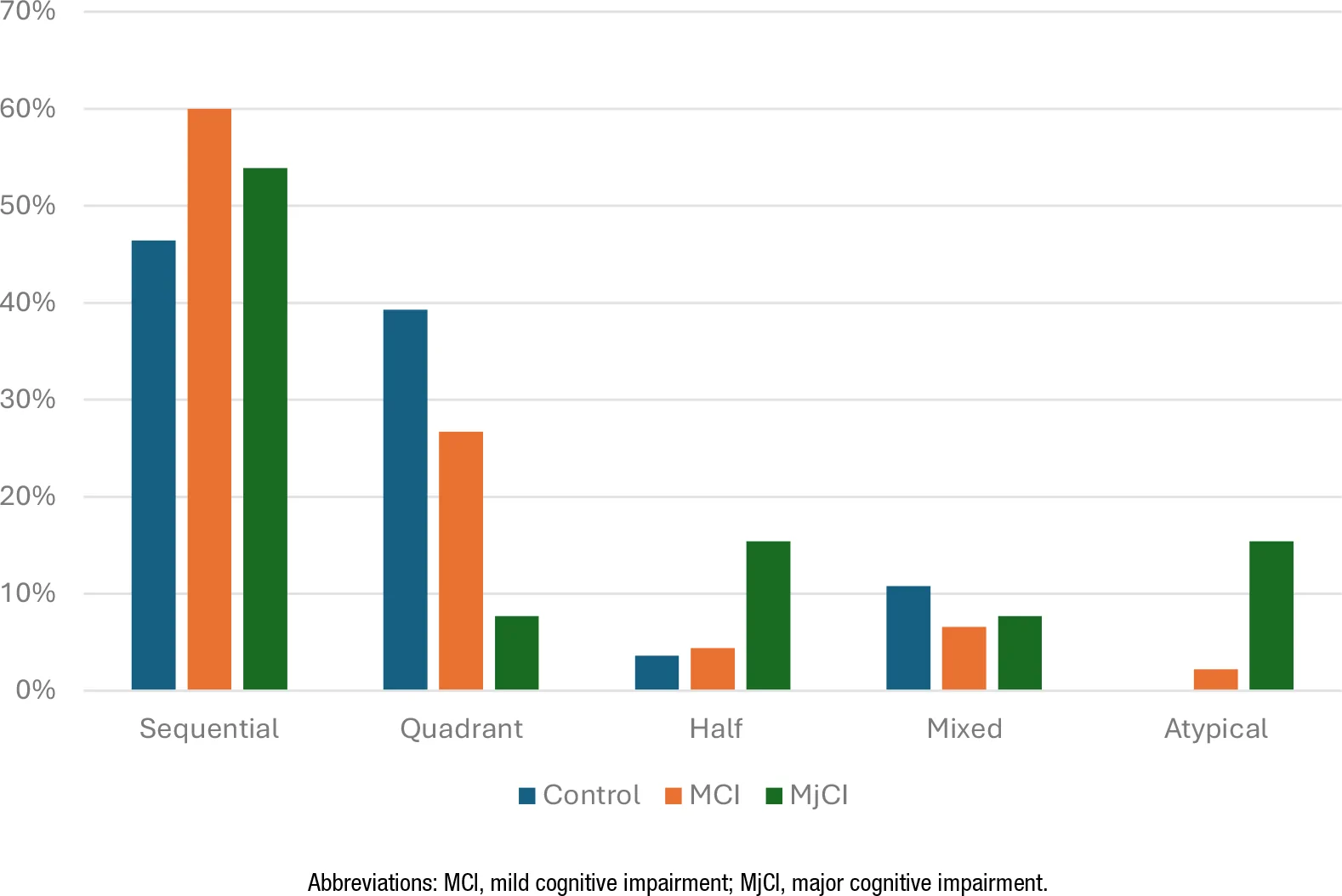
Distribution ratio of numerical sequence strategies.

Abbreviations: MCI, mild cognitive impairment; MjCI, major cognitive impairment.

Considering the Numerical Sequence (Table 3), Sequential is the most frequent of the three groups. The frequency of Quadrant decreases. Half and Atypical increase their occurrence as cognitive impairment intensifies (Figure 3).

**Table 3:** Discrepancy between observed and expected frequency of association between Clock Drawing Test Strategies — Numerical Sequence and neuropsychological profile.

n	Frequency	Control	MCI	MjCI
		28	45	13
Numerical sequence				
Sequential	Observed	13	27	7
	Expected	15.3	24.6	7.1
Quadrant	Observed	11	12	1
	Expected	7.8	12.6	3.6
Half	Observed	1	2	2
	Expected	1.6	2.6	0.8
Mixed	Observed	3	3	1
	Expected	2.3	3.7	1.1
Atypical	Observed	0	1	2
	Expected	1	1.6	0.5

Abbreviations: MCI, mild cognitive impairment; MjCI, major cognitive impairment.

Analysis of the subtypes of the MCI showed the occurrence of the General Sequence circle-number-center-hand (Table 4) and the Sequential Numerical Sequence (Table 5) greater than expected in the MCI-A, and lower than expected in groups MCI-A MD and MCI N-A. As the number of participants was reduced, several occurrences were fewer than 5. Atypical General Sequence showed a significant correlation with cognitive impairment (X(2)=10.043, p<0.05).

**Table 4:** Discrepancy between observed and expected frequency of association between Clock Drawing Test Strategies — General Sequence and mild cognitive impairment subtypes.

n	Frequency	MCI-A	MCI-A MD	MCI N-A
		18	17	10
General sequence				
Circle-number-center-hand	Observed	7	5	3
	Expected	6	5.7	3.3
Circle-number-hand	Observed	1	6	2
	Expected	3.6	3.4	2
Circle-center-number-hand	Observed	2	3	2
	Expected	2.8	2.6	1.6
Atypical	Observed	2	1	1
	Expected	1.6	1.5	0.9
Mixed	Observed	4	1	2
	Expected	2.8	2.6	1.6

Abbreviations: MCI-A, Amnestic MCI-Single Domain; MCI-A MD, Amnestic MCI-Multiple Domain; MCI N-A, Non-Amnestic MCI Single Domain.

**Table 5:** Discrepancy between observed and expected frequency of association between Clock Drawing Test Strategies — Numerical Sequence and mild cognitive impairment (subtypes.

n	Frequency	MCI-A	MCI-A MD	MCI N-A
		18	17	10
Numerical sequence				
Sequential	Observed	13	9	5
	Expected	10.8	10.2	6
Quadrant	Observed	4	4	4
	Expected	4.8	4.5	2.7
Half	Observed	1	0	1
	Expected	0.8	0.8	0.4
Mixed	Observed	0	3	0
	Expected	1.2	1.1	0.7
Atypical	Observed	0	1	0
	Expected	0.4	0.4	0.2

Abbreviations: MCI-A, Amnestic MCI-Single Domain; MCI-A MD, Amnestic MCI-Multiple Domain; MCI N-A, Non-Amnestic MCI Single Domain.

## DISCUSSION

Although CDT is a widely used test, analog clocks are less common among younger people in this digital era^25^. Studies in recent years suggest that professionals are less familiar with the CDT^26,27^, which may reduce its usefulness and validity in the coming decades^7^. However, this has not affected the number of studies that used CDT, validating its clinical use^28^. Contemporary process-based assessment tools and digital analysis of CDT are also frequent, but the use of paper-and-pen methods is very important in clinical and research contexts due to their easy and accessible administration. Therefore, deepening research into traditional methods remains necessary.

The combined use of semi-quantitative/quantitative and qualitative scoring systems is recommended for the evaluation of CDT^2,12^. However, analysis of the clock drawing process and sequential aspects of executive functioning was only possible through analysis by the CDT Strategies scoring system. CDT strategies used by older adults with cognitive impairment were described and classified. In comparison to the strategies used by the individuals without cognitive impairment, relevant results could be described. Subcategories reflecting stronger planning and organizational strategies decrease with increasing cognitive impairment, while inefficient strategies become more frequent.

### Clock drawing test strategies

General Sequence strategies proved to be relevant measures for planning evaluation, with better indices than the Numerical Sequence. More specifically, the Circle-number-center-hand is the General Sequence strategy that has had the best performance in planning and organization^2^. This strategy presented a higher frequency than expected in the controls, and its frequency decreased with impairment progression. Since the Circle-number-center-hand strategy is associated with better performance in executive functioning, it can be assumed that as cognitive impairment intensifies, executive function is impaired^29,30^. Planning and sequencing are some of the skills that may be most compromised as people age^30^. Difficulties in performing the CDT may be related to poor planning for patients with MCI^31^. This CDT construction profile, in AD patients, can be attributed to characteristics of the pathology, such as impairment in visuospatial functioning (right parietal lobe) and more specifically in visuospatial planning (frontal lobe)^32,33^.

Conversely, Atypical General Sequence Strategy has a lower-than-expected occurrence in MCI and controls and a higher-than-expected occurrence in MjCI. Considering that this strategy is associated with worse performance in planning and organization^2,11^, it is reinforced that as the level of cognitive impairment intensifies, the ability to plan and organize is even more impaired.

Impairment in functionality is a criterion for the diagnosis of MjCI^34^, as it is a predictor of loss of functionality^35^. The greater presence of the Atypical General Sequence in this group is justified by the correlation of executive performance with functionality^36^.

Functional decline and its correlation with behavioral markers and physical activity patterns are being widely studied^37,38,39^, but still unclear^40^. Scales focused on the capacity of the individual to accomplish specific functional tasks and conventional physical activity metrics alone have limited ability to predict physical function impairment due to multidimensional features^38^. In view of this, this new proposed scoring method of CDT, together with other measures, may contribute to the prediction of functional decline and timely intervention.

Analysis of the MCI subtypes showed that MCI-A had a higher occurrence of General Sequence Circle-number-center-hand, previously associated with better executive performance^2,11^, than the other subtypes (MCI-A MD and MCI N-A). However, MCI-A also had a greater association with the Numerical Sequence Sequential, which is associated with lower executive functioning performance. This mixed pattern of responses for MCI subtypes is justified since it is considered a heterogeneous entity^18,41^.

The results analysis of Numerical Sequence Strategies also showed a correspondence with the literature regarding the impairment of executive functioning in MjCI^32,42^. It was previously found that the Quadrant subcategory was the most associated with measures of better executive functioning^2^. This standard of numerical structuring for clock drawing and its correlation with better executive functioning was also verified in another study that evaluated digital CDT. The authors also correlated this type of construction strategy with better efficiency in information organization and planning, correlating neuropsychological data and neuroimaging exams^10^.

In the present study, the Quadrant strategy had a higher-than-expected occurrence among controls and a lower-than-expected occurrence in the MjCI. As mentioned above, MjCI has poor performance in executive functioning^32,42^, thus has a lower frequency of the Quadrant strategy.

On the other hand, the Sequential strategy, which was associated with low executive performance^2^, presented a higher frequency than expected in the MCI group and a lower frequency than expected among controls. Again, these results confirm that CDT Strategies scoring system showed coherence with the literature^43^. After all, the group without cognitive impairment had an association with better performance in executive functioning, while the group with MCI was associated with impairment of this function^31,34^.

**Figure 4:**
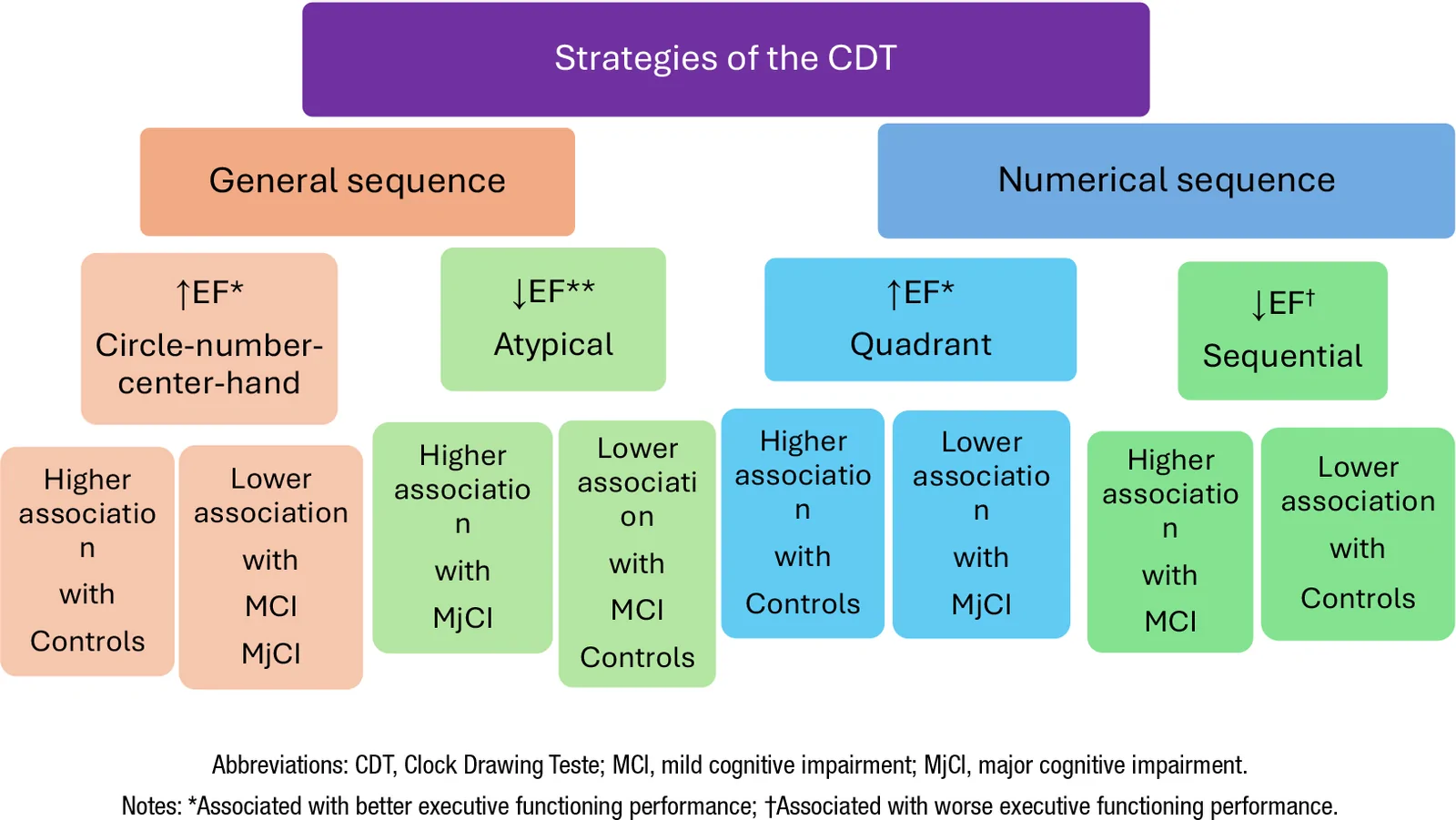
Systematization of the associations between the level of cognitive impairment and the Clock Drawing Teste planning strategies.

Abbreviations: CDT, Clock Drawing Teste; MCI, mild cognitive impairment; MjCI, major cognitive impairment.

Notes: *Associated with better executive functioning performance; †Associated with worse executive functioning performance.

The qualitative analysis of the data was possible through frequency distribution and has relevance and clinical utility for more specific descriptions of neuropsychological profiles. There was a significant association between the Atypical General Sequence and the different levels of cognitive impairment. This aspect can be justified by the fact that the sample studied was reduced in comparison with the number of categories present in the new system, as can be seen in Figure 4.

Compared to semi-quantitative and qualitative systems, the CDT Strategies scoring system opens the possibility of observing the CDT construction entire process, thus categorizing the sequence and making it possible to describe, in detail, the older adult’s executive functioning characteristics, especially planning and organization. It is observed that the qualitative information that the system offers is important to analyze the neuropsychological profile of older adults. Therefore, it contributes as an aid to the clinical diagnosis of cognitive impairment.

Cognitive impairment can be manifested through different behavioral markers, and its study may help clinicians with MCI screening^39^. This strategy-based method proposes to analyze sequencing behaviors and expand the use of CDT to help cognitive impairment screening.

Even so, behavioral markers and the overall analysis of the sequencing process of the drawing could also be extended to other types of drawing-based cognitive tests. This descriptive approach may access behavioral markers, expanding perspectives through the diagnosis of cognitive impairment. After all, both cognitive and functional impairment present themselves in a multidimensional way.

The structured planning strategies decrease, and atypical strategies increase with worsening cognitive impairment, appearing largely consistent with established literature on executive dysfunction in MCI and dementia. Studies of the CDT and related executive measures have shown that individuals with MCI exhibit reduced strategic organization and increased planning errors, representing an intermediate profile between normal aging and dementia^8,44^. In dementia, disorganized spatial layout, stimulus-bound responding, and conceptual errors become increasingly prevalent and correlate with the severity of executive dysfunction^1,45^. These qualitative changes reflect a deterioration of frontal-executive control systems responsible for planning, sequencing, monitoring, and cognitive flexibility^46^, and have been associated with functional decline and loss of independence in everyday activities^47^.

The advantage of the heterogeneity of the sample was the wide range of neuropsychological profiles presented. Thus, the role of organization and planning strategies in cognitive impairment was verified. Different types of planning strategies for different profiles of cognitive impairment were highlighted, enabling greater applicability to clinical approaches.

Age and years of formal education are important participant characteristics in studies with older adults, as increasing age and low education are risk factors for the development of MjCI^25^. The sample stands out with a high level of education, and the groups did not significantly differ in age. On the other hand, comparing the age group, the mean age of MjCI is higher when compared to the control and MCI. Advanced age has also been associated with modest declines in CDT performance, even among cognitively healthy individuals. Age-related changes in processing speed, visuospatial abilities, and executive functioning may affect clock organization, number placement, and hand positioning. Consequently, older adults may obtain lower scores irrespective of pathological cognitive decline^1,26^. Educational attainment has a strong impact on CDT outcomes. Individuals with lower levels of formal education or limited literacy may perform poorly due to reduced familiarity with analog clocks, less developed visuoconstructive skills, or limited experience with structured testing. In contrast, higher education is associated with better performance and may reflect greater cognitive reserve^27^.

The contribution of the present study goes beyond its application to clock drawing, as it provides a discussion of planning strategies during the drawing process^48^, extending the discussion to future studies with different types of drawings.

Finally, the observed pattern of CDT performance is consistent with broader evidence on executive dysfunction, and can be extrapolated to other cognitive tasks. Executive functioning represents a domain-general control system that supports planning, sequencing, monitoring, cognitive flexibility, and goal-directed behavior across multiple neuropsychological activities. Declines in these processes affect performance not only on CDT but also on tasks such as the Trail Making Test (Part B), verbal fluency tests, and complex instrumental activities of daily living^46,49^. Because CDT requires strategic planning, visuoconstruction, inhibition, and self-monitoring, deterioration of planning strategies likely reflects generalized disruption of executive control mechanisms. Qualitative aspects of CDT performance — such as organizational approach, sequencing errors, stimulus-bound responses, and planning inefficiencies — have been associated with executive dysfunction and correlate with performance on other executive measures^1,44^. Similarly, reduced strategic organization parallels findings in verbal fluency tasks, where diminished clustering and switching reflect impaired cognitive flexibility and search strategies^50^. Theprogressive shift from efficient to inefficient strategies may also indicate increasing difficulty in everyday functioning. Executive deficits affect complex activities such as medication management, financial decision-making, meal preparation, and navigation, all of which depend on planning, sequencing, and error monitoring^47^. Thus, qualitative CDT changes may serve as behavioral markers of broader functional decline. Taken together, qualitative analysis of CDT planning strategies provides information that extends beyond a single screening measure. Changes in strategy use may reflect global executive system deterioration and help anticipate impairments across other neuropsychological domains and functional abilities.

This study has some limitations, such as modest sample size, high educational level, and heterogeneity within clinical groups. The cross-sectional nature of the analysis and the use of a paper-and-pen framework screening test may also be boundaries within the context of current automated analysis and wearable devices. Thus, for future studies, the evaluation of possible correlations between CDT Strategies and other behavioral markers and physical activity patterns is suggested, in addition to the development of standardization in groups stratified by age and education level with larger sample size.

## Data Availability

The datasets generated and/or analyzed during the current study are publicly available at DBD PUC-Rio Pergamum 1712308_2020_completo.pdf
